# Unlocking the Therapeutic Symphony: A Systematic Review Exploring the Role of Levosimendan in the Management of Heart Failure

**DOI:** 10.7759/cureus.62799

**Published:** 2024-06-20

**Authors:** Rahmat Ali, Waseem Sajjad, Iqra Mushtaq, Humaira Farooqi, Amir Sohail, Hamza Wazir Khan, Pakeezah Tabasum, Abdul Haseeb, Wajahat Ullah Ismail

**Affiliations:** 1 Department of Medicine, Mayo Hospital, Lahore, PAK; 2 Department of Cardiology, Guy's and St Thomas' NHS Foundation Trust, London, GBR; 3 Department of Pathology, Mayo Hospital, Lahore, PAK; 4 Department of Medicine, Peoples University of Medical and Health Sciences for Women, Nawabshah, PAK

**Keywords:** cardiology, right ventricular dysfunction, cardiomyopathy, left ventricular ejection fraction, calcium sensitizing inotrope, heart failure, levosimendan

## Abstract

Levosimendan, a novel drug, a calcium-sensitizing inotrope, has emerged as a potential therapeutic modulator for heart failure (HF). This review appraises the efficacy and safety of levosimendan in managing HF, in different clinical settings. The study aims to examine the clinical outcomes reported in the selected trials to determine the effectiveness of levosimendan in improving key parameters related to HF.

Seven relevant studies encompassing 1200 participants were identified from three databases. Inclusion criteria included clinical trials that investigated the therapeutic efficacy of levosimendan in the treatment of HF, and studies involving both adult and pediatric participants. Exclusion criteria involved studies with insufficient data, studies other than clinical trials, case reports, letters to the editor, conference papers, grey literature, and studies published in a language other than English.

Upon evaluating the included studies, it was found that levosimendan shows improved hemodynamics and clinical efficacy in patients with severe septic cardiomyopathy. Levosimendan enhanced right ventricular (RV) function in patients with RV dysfunction after mitral valve (MV) surgeries and decreased the amount of N-terminal pro-B-type natriuretic peptide (NT-ProBNP) in non-ST elevated myocardial infarction (NSTEMI) patients with elevated NT-proBNP, all without increasing the overall cost or duration of hospitalization.

Despite variations in study designs and participant characteristics, evidence suggests levosimendan significantly improves left ventricular ejection fraction (LVEF) and exercise tolerance measured by a six-minute walk distance. Notably, its safety profile appears favorable with minimal arrhythmic events and comparable rates of adverse effects to a placebo. This systematic review highlights levosimendan’s promising potential for HF management, warranting further research to solidify its clinical role.

## Introduction and background

Heart failure (HF) remains a debilitating and prevalent global health burden, affecting over 64 million people worldwide [1]. Current therapeutic options for HF often fall short despite significant advancements in medical management. They fail to address the complex pathophysiology and multifaceted symptoms of the disease adequately [2]. This unmet clinical need underscores the urgent search for novel, effective, and potentially transformative treatment strategies.

Levosimendan, a unique calcium-sensitizing inotrope, has proven to be a promising candidate in the HF treatment landscape. Levosimendan binds to cardiac troponin C and enhances the contractile response to calcium, offering a distinct mechanism of action compared to traditional inotropes [3]. This has several potential advantages, including improved cardiac output without affecting myocardial oxygen consumption, a reduction in pulmonary congestion and dyspnea, and potentially favorable neurohormonal effects [4,5].

Early clinical trials and preclinical studies suggested levosimendan’s therapeutic potential; it has considerably expanded the landscape of evidence in recent years. A plethora of clinical trials examining the safety and effectiveness of levosimendan across various HF etiologies and patient populations have been published in 2023, particularly. However, following this burgeoning body of research and synthesizing the latest funding for clinical practice can be challenging. Therefore, this review aims to comprehensively evaluate the efficacy and safety of levosimendan for HF treatment in different patient scenarios. The primary objective of this systematic review is to comprehensively determine and synthesize the pre-existing evidence from clinical trials published in recent years, examining the therapeutic efficacy of levosimendan in the treatment of HF. This study aims to determine the effectiveness of levosimendan by examining the clinical outcomes reported in selected trials i.e. to find out the improvement of key parameters related to HF, including but not limited to ejection fraction, exercise tolerance, and symptom relief. This also analyzes the different patient populations included in clinical trials to identify the subgroups that may obtain particular benefits from levosimendan, considering factors such as age, gender, HF severity, and comorbidities. This review also aims to assess the incidence and nature of adverse events associated with levosimendan treatment in patients with HF and to determine the safety data from selected trials. Along with this, this review clarifies inconsistencies and discrepancies to provide a more cohesive understanding of the therapeutic effects of levosimendan on HF.

In short, this review focuses on the summarization of the findings from selected trials and offers a comprehensive overview of the current state of knowledge regarding the role of levosimendan in the treatment of HF. This synthesis will guide us to future research directions and informed clinical practice, contributing to the ongoing dialogue on optimal treatment strategies for HF management.

## Review

Materials and methods

Search Result

A comprehensive search was employed to ensure the complete coverage of published literature. The search strategy involved a rigorous exploration of three electronic databases such as PubMed, Embase, and Google Scholar. Levosimendan and HF-related medical subject headings (MeSH) were combined with keywords for literature search. Variants of “Levosimendan” and “Heart failure” and other synonym phrases were included in the search terms. To hone the search results and guarantee that only possibly relevant researches were retrieved, the Boolean Operators AND and OR were utilized.

Study Selection

We identified 189 potentially relevant studies from the aforementioned databases. After removing redundant studies, the titles and abstracts of 153 retrieved articles were screened by two independent reviewers to check the relevance and reliability of studies on predefined inclusion and exclusion criteria. A third independent reviewer was employed to settle any dispute among reviewers. After scrutiny, seven articles exactly meeting our criteria were included in the systematic review.

Inclusion and Exclusion Criteria

Inclusion criteria included clinical trials that investigated the therapeutic efficacy of levosimendan in the treatment of HF, studies involving both adult and pediatric participants, clinical trials reporting diverse patient populations, ranging from different severities of HF, studies reporting primary clinical outcomes related to levosimendan’s impact on HF, studies published in peer-reviewed journals and studies published in the English language. Exclusion criteria involved studies with insufficient data, studies other than clinical trials, case reports, letters to the editor, conference papers, grey literature, and studies published in a language other than English.

**Figure 1:**
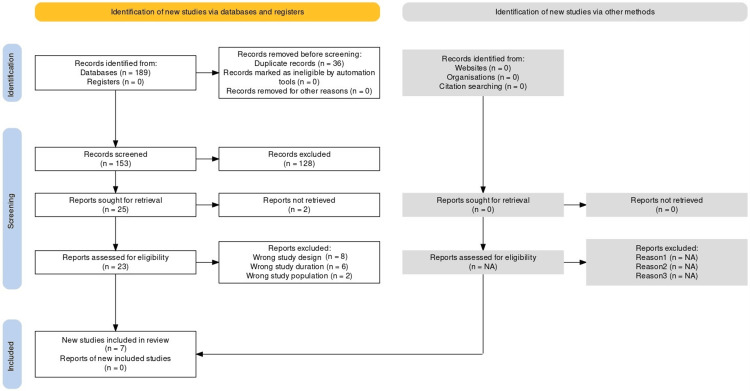
PRISMA flowchart PRISMA: Preferred Reporting Items for Systematic Reviews and Meta-Analyses

To ensure consistent and clear reporting, a standardized data extraction sheet was used to report key information from each study, including its objectives, participant details, interventions, measurements, primary findings, and overall conclusions. The study selection process was guided by the Preferred Reporting Items for Systematic Reviews and Meta-Analyses (PRISMA) guidelines, which promote transparency and reproducibility in systematic reviews and meta-analyses. A PRISMA flow diagram summarizing the study selection process is presented in Figure 1.

Quality Assessment

**Figure 2:**
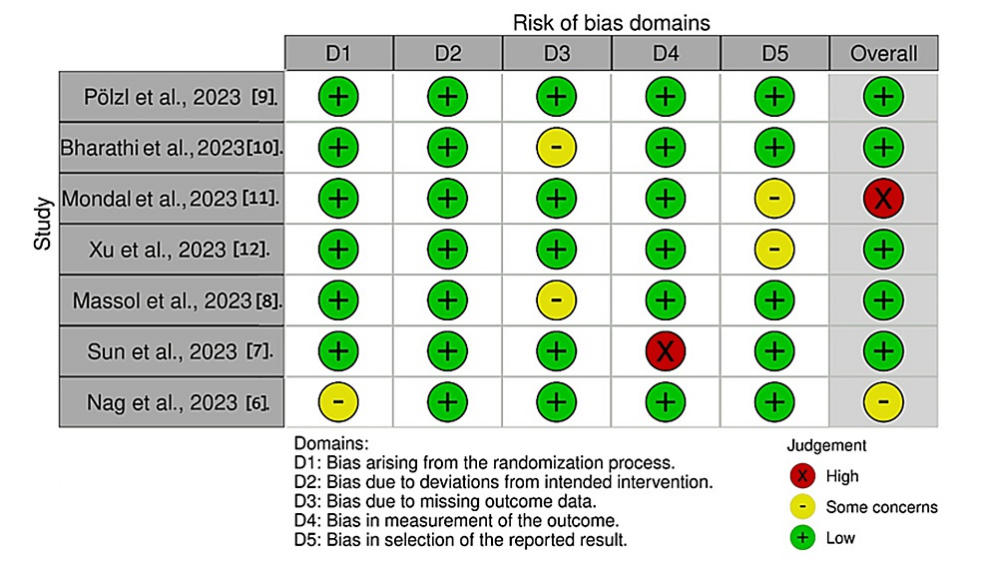
Risk of bias assessment of randomized controlled trials (traffic light plot)

**Figure 3:**
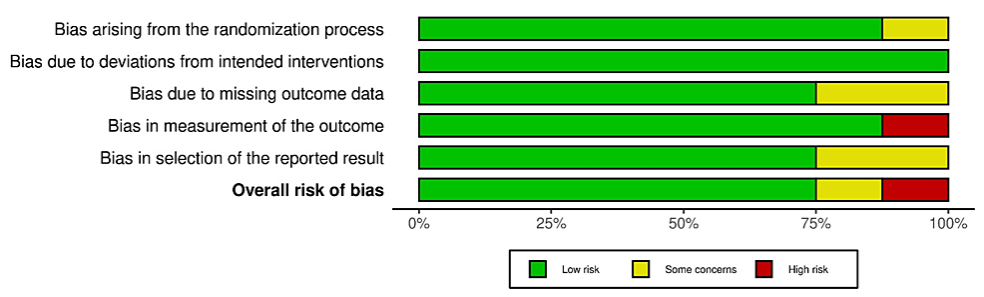
Risk of bias assessment of randomized controlled trials (summary plot)

Risk of Bias 2 (RoB 2) guided a thorough evaluation of the RoB in the included studies. Two independent reviewers examined key areas of RoB like randomization, intervention adherence, missing data, and outcome measurement, tailoring their approach to account for the randomized controlled trials. The traffic light plot and summary plots for RoB of included studies are given in Figures 2-3.

Results

Comparing Levosimendan and Milrinone in Ventricular Septal Defect (VSD) Closure With Pulmonary Arterial Hypertension (PAH)

Levosimendan and milrinone had similar effects on the prevention of low-cardiac-output syndrome (LCOS) in patients undergoing surgical closure of VSD with PAH. Levosimendan was associated with lower mean arterial pressure, longer duration of ventilation, and postoperative intensive care unit (ICU) stay, and no difference in the myocardial performance index of the left or right ventricle compared to milrinone. There were two in-hospital deaths, one in each group [6].

Levosimendan vs Dobutamine in Severe Septic Cardiomyopathy

Patients with severe septic cardiomyopathy responded better to levosimendan than to dobutamine in terms of improved hemodynamics and clinical efficacy. In comparison to dobutamine, levosimendan shortened the duration of mechanical ventilation, raised cardiac index (CI), left ventricular ejection fraction (LVEF), stroke volume index (SVI), and fluid volume, and lowered the dose of norepinephrine and cardiac troponin I. The length of stay in the ICU, the cost of ICU therapy, and the 28-day mortality did not differ significantly across the groups [7].

Levosimendan in Postcardiotomy Cardiogenic Shock

Levosimendan did not improve extracorporeal membrane oxygenation (ECMO) weaning or death rate in patients with postcardiotomy cardiogenic shock. Levosimendan had no effect on pulmonary capillary wedge pressure (PCWP), E/e’ ratio, or 30-day and one-year mortality compared to no levosimendan. The control group results in four cardiac deaths during hospitalization. Levosimendan and no levosimendan had similar hospitalization costs and lengths [8].

Post-hospitalization Stability in HF With Reduced LVEF

Levosimendan did not improve post-hospitalization clinical stability in patients with HF and reduced LVEF. Levosimendan had no effect on the global rank score, the primary endpoint, but increased the incidence of death, rehospitalization, or worsening HF, the individual components of the primary endpoint, compared to placebo. There were no significant differences in six-month survival, hospitalization cost, or length between the groups [9].

Levosimendan in Right Ventricular (RV) Dysfunction During Mitral Valve (MV) Surgeries

Levosimendan improved RV function in patients with RV dysfunction undergoing MV surgeries. Levosimendan increased RV size, inferior vena cava (IVC) diameter, right ventricular fractional area change (RVFAC), tricuspid annular plane systolic excursion (TAPSE), and systolic pulmonary artery pressure (SPAP) compared to placebo at 24 hours and seventh day postoperatively. There were no significant differences in major adverse cardiovascular events (MACE), hospitalization cost, or length between the groups [10].

Comparing Levosimendan and Milrinone in Off-Pump Coronary Artery Bypass (OPCAB)

Levosimendan reduced left atrial pressure (LAP) better than milrinone in patients undergoing OPCAB. Levosimendan decreased PCWP and E/e’ ratio more than milrinone at different stages of OPCAB. There were no considerable differences in MACE, hospitalization cost, or length between the groups [11].

Levosimendan in Non-ST Elevated Myocardial Infarction (NSTEMI) Patients With Elevated N-terminal Pro-B-Type Natriuretic Peptide (NT-ProBNP)

In NSTEMI patients with elevated NT-proBNP, levosimendan decreased the level of NT-proBNP without extending hospital stay or expenses, but it had no discernible effect on MACE at six months or during hospitalization. Comparing levosimendan to no levosimendan showed no difference in 30-day and one-year mortality, PCWP, or E/e' ratio. During hospitalization, there were four cardiac fatalities in the control group. No adverse effects or serious adverse effects were reported in both groups during levosimendan treatment [12].

Discussions

The role of levosimendan as a therapeutic agent in various cardiovascular conditions remains complex and nuanced, with studies illuminating both promising and conflicting results. This discussion aims to synthesize the findings presented from several studies, addressing the potential benefits and limitations of levosimendan across various clinical settings.

In patients, undergoing surgical closure of VSD with PAH, levosimendan and milrinone demonstrated comparable efficacy in preventing LCOS [13]. While both drugs improved hemodynamics, levosimendan was associated with lowered mean arterial pressure undergoing, potentially highlighting its vasodilatory effect. However, this benefit was offset by longer ventilation and ICU stays and suggests potential drawbacks in postoperative recovery. Overall, the choice between levosimendan and milrinone in this context may depend on individual patient factors and risk tolerance.

In contrast, levosimendan emerged as superior to dobutamine in managing severe septic cardiomyopathy [14]. Levosimendan decreases the requirement of norepinephrine and cardiac troponin I levels and significantly improves CI, LVEF, SVI, and fluid volume. These findings indicate its potential to enhance myocardial contractility and reduce stress on the failing heart in septic shock. Notably, levosimendan also shortened mechanical ventilation duration, suggesting beneficial effects on respiratory functions. Despite these benefits, levosimendan did not affect treatment costs, length of stay in ICUs, or 28-day mortality, underscoring the intricate relationship between hemodynamic improvement and clinical outcomes in this critically sick sample.

The picture becomes less clear when examining levosimendan’s role in post-cardiotomy cardiogenic shock [15]. In this study, levosimendan failed to improve ECMO weaning or mortality compared to the control group. Additionally, it showed no impact on PCWP, E/e' ratio, or long-term survival. These findings suggest that levosimendan may have limited usage in this specific setting, possibly due to the complex pathophysiology of post-surgical myocardial dysfunction.

The results from the study suggested highlighting the need for cautious interpretation of levosimendan potential in patients with HF and reduced LVEF [16]. Although levosimendan did not improve the primary outcome of a composite score encompassing death, rehospitalization, or worsening HF, it individually increased the incidence of each component. This seemingly paradoxical finding raises concerns about potential adverse effects associated with long-term levosimendan use in this population. The lack of impact on survival, hospitalization cost, or length further underscores the need for larger, long-term trials to definitely establish its role in HF management.

Despite these mixed results in broader settings, levosimendan demonstrated beneficial effects in specific surgical contexts. In patients undergoing MV surgery with RV dysfunction, levosimendan improved RV function, as evidenced by increased RV size, IVC diameter, RVFAC, TAPSE, and SPAP [17]. These findings suggest its potential to support RV function during MV surgery, potentially lowering postoperative complications. Similarly, levosimendan outperformed milrinone in reducing LAP during OPCAB surgery [18]. This vasodilatory effect may contribute to improved hemodynamic stability and potentially optimize surgical outcomes.

It is crucial to talk about levosimendan use in non-surgical situations. Levosimendan successfully reduced NT-proBNP in patients of NSTEMI who had elevated NT-proBNP levels, but failed to significantly improve MACE or other clinical outcomes [13]. Interestingly, it did not increase hospitalization costs or length, suggesting potential economic benefits despite modest clinical effects. The lack of adverse events associated with levosimendan treatment access studies adds to its safety profile [19].

Like any other scientific study, our review also carries some limitations that need to be acknowledged before using its findings. The reviewed studies vary in patient populations and endpoints, making direct comparisons and definitive conclusions challenging. Some studies have small sample sizes, reducing their generalizability and statistical power to detect significant differences. Most studies focus on short-term outcomes, leaving the long-term efficacy and safety of levosimendan unclear. The studies mainly assess levosimendan in a specified context, limiting our understanding of its broader applicability across diverse cardiovascular conditions. While hospitalization costs were mentioned in some studies, a comprehensive cost-effectiveness analysis considering long-term outcomes and resource utilization is missing.

Future directions

Robust trials with larger and diverse patient populations are needed to confirm or refute the observed benefits and harms of levosimendan across various cardiovascular conditions. Longer follow-up periods are essential for assessing levosimendan’s long-term safety and efficacy, especially in chronic illnesses like HF. Investigating the effects of levosimendan based on specific patient characteristics, such as severity of illness, comorbidities, and baseline hemodynamic profiles, may help identify patient populations who benefit most from the treatment. Conducting comprehensive cost-effectiveness analyses is essential to determine the feasibility and value proposition of levosimendan compared to existing treatment options. Examining the potential benefits and synergy of combining levosimendan with other established or novel cardiovascular medications could lead to more effective treatment strategies.

## Conclusions

Levosimendan's therapeutic potential remains an intriguing yet intricate proposition. While it demonstrates valuable benefits in specific settings, such as septic cardiomyopathy and surgical contexts involving RV dysfunction or OPCAB, its efficacy in broader applications requires further investigation. The mixed results in HF and postcardiotomy shock highlight the need for careful patient selection and consideration of potential downsides. Future research may explore a more defined role in specified conditions.
